# Multi-Step Combined Upfront Surgery for Locally Advanced Paravertebral Sarcoma: A Case Report

**DOI:** 10.3389/fsurg.2021.664089

**Published:** 2021-04-26

**Authors:** Umberto Cariboni, Nicolò Gennaro, Francesco Costa, Salvatore Lorenzo Renne, Pierluigi Novellis, Andrea Marrari, Alexia Francesca Bertuzzi, Efrem Civilini

**Affiliations:** ^1^Division of Thoracic Surgery, Humanitas Clinical and Research Center–Istituto di Ricovero e Cura a Carattere Scientifico (IRCCS), Milan, Italy; ^2^Department of Radiology, Humanitas Clinical and Research Center–Istituto di Ricovero e Cura a Carattere Scientifico (IRCCS), Milan, Italy; ^3^Department of Biomedical Sciences, Humanitas University, Milan, Italy; ^4^Division of Neurosurgery, Humanitas Clinical and Research Center–Istituto di Ricovero e Cura a Carattere Scientifico (IRCCS), Milan, Italy; ^5^Department of Pathology, Humanitas Clinical and Research Center–Istituto di Ricovero e Cura a Carattere Scientifico (IRCCS), Milan, Italy; ^6^Division of Thoracic Surgery, San Raffaele Scientific Institute–Istituto di Ricovero e Cura a Carattere Scientifico (IRCCS), Milan, Italy; ^7^Department of Medical Oncology & Hematology–Humanitas Clinical and Research Center–Istituto di Ricovero e Cura a Carattere Scientifico (IRCCS), Milan, Italy; ^8^Division of Vascular Surgery, Humanitas Clinical and Research Center–Istituto di Ricovero e Cura a Carattere Scientifico (IRCCS), Milan, Italy

**Keywords:** sarcoma, multidisciplinary (treatment), UPS, surgery, aorta–thoracic, vertebra bone

## Abstract

**Background:** Paravertebral localization of primary undifferentiated pleomorphic sarcoma (UPS) with bone and vascular involvement is infrequent and challenging. Multi-step surgical procedure has been described as a feasible and effective option to achieve sustained local tumor control.

**Methods:** We report on a 62-year old man with paravertebral UPS infiltrating the aortic wall and the 9th thoracic vertebra who underwent a multi-step surgical procedure aimed at achieving oncologic radicality through a coordinated effort between thoracic, vascular and spinal surgeons. After balancing the risks and benefits of perioperative therapies, upfront surgery was performed including aortic resection with bypass grafting followed by a triple *en bloc* vertebrectomy with tumor excision. Mid-term follow-up (22 months) is then provided.

**Results:** The combined procedure achieved oncological radicality and no local recurrence in the mid-term. No major complications occurred.

**Conclusions:** Multi-step and multi-specialty surgery is a feasible and effective strategy to treat primary UPS in unfavorable localization. A strategic cooperation between surgeons and a multidisciplinary tumor board is required to define an optimal, personalized treatment strategy in sarcoma patients.

## Background

Previously known as malignant fibrous histiocytoma, UPS is a rare high-grade mesenchymal neoplasm that may arise ubiquitously (1). Radical surgery is the cornerstone of the treatment of localized soft tissue sarcoma (STS), with perioperative treatments being offered in selected histotypes and clinical presentations (2). Being characterized by an aggressive behavior, a multi-specialty surgery may be necessary to treat locally-advanced STS involving several structures such as the bone, vessels and nerves (3).

In this single case report, we describe a challenging multi-step upfront surgery aimed at providing oncologic radicality in a patient with locally-advanced paravertebral UPS.

## Methods

**Figure 1:**
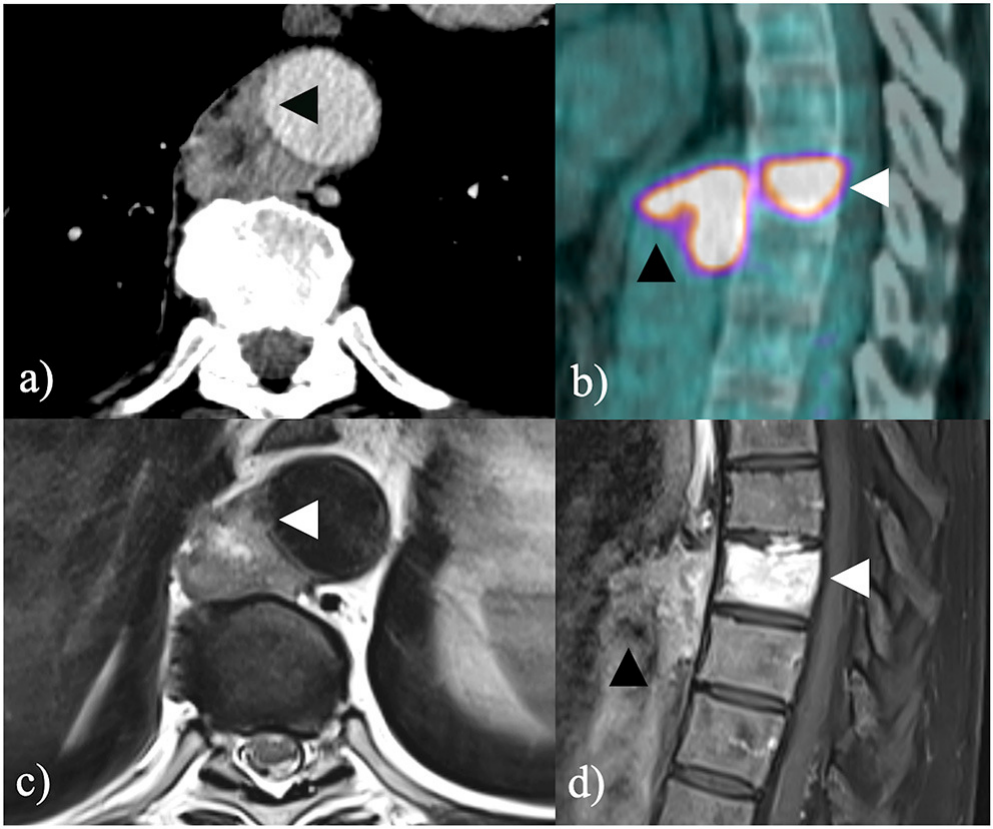
**(a)** Contrast-enhanced CT scan shows a paravertebral soft tissue mass encasing the thoracic descending aorta with suspicious infiltration of the aortic wall (black arrowhead); **(b)** further ^18^F-FDG PET/CT shows intense radiopharmaceutical uptake both within the soft tissue tumor (black arrowhead) and within the 9th thoracic vertebra (white arrowhead); **(c)** contrast-enhanced MRI T1-weighted image confirms infiltration of the aortic wall (white arrowhead) and **(d)** heterogeneous contrast enhancement both within the soft tissue tumor (black arrowhead) and within the 9th thoracic vertebra (white arrowhead).

A 62-year-old man presented with abdominal pain in suspected kidney stone disease. The patient underwent a multiphasic abdominal CT scan which confirmed urolithiasis and showed a 25 × 19 mm paravertebral soft tissue lesion anterior to 9th thoracic vertebrae (T9), encasing the descending thoracic aorta (Figure 1a). Notably, the patient reported no neurological symptoms.

Spinal magnetic resonance imaging (MRI) confirmed an extensive T9 bone involvement and a focal infiltration of the aortic wall (Figures 1c,d). 18-fluorodeoxyglucose positron emission tomography/computed tomography (^18^F-FDG-PET/CT) showed intense radiopharmaceutical uptake and excluded distant metastases (Figure 1b). A CT-guided percutaneous core-needle biopsy was warranted and histology described undifferentiated pleomorphic sarcoma (UPS). The case was extensively discussed in a multidisciplinary tumor board (MDT) involving thoracic, spinal and vascular surgeons along with medical and radiation oncologists, radiologists and pathologists with expertise in rare soft tissue and bone tumors. After a comprehensive assessment of the risks and benefits of effective therapeutic options, the MDT agreed to perform a wide upfront surgery aimed at achieving oncological radicality. In particular, a multi-step surgical approach including segmental resection of the descending thoracic aorta followed by *en bloc* removal of T8-T9-T10 and adjuvant sequential chemo- and radiotherapy were planned.

**Figure 2:**
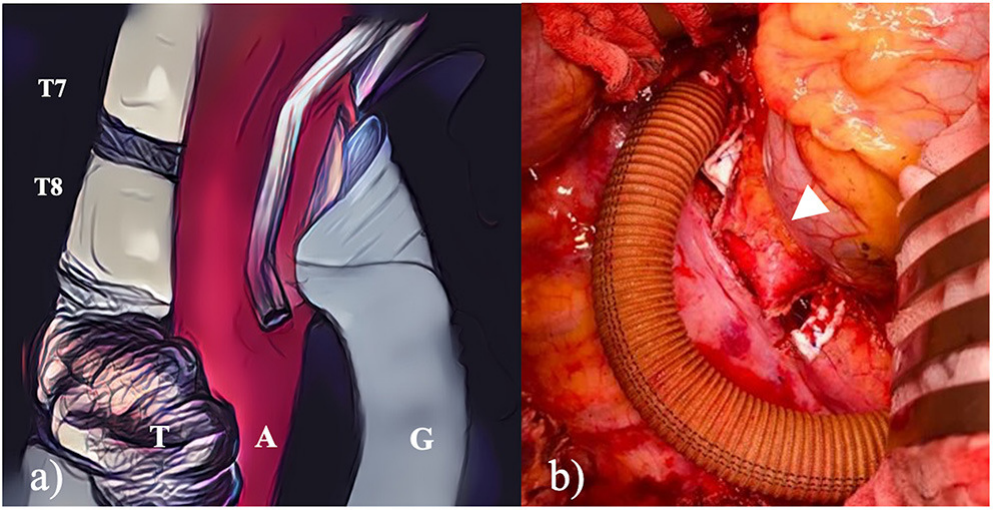
**(a)** The scheme detailed the side-biting clamp that allowed the aortic graft to be anatomised without interrupting peripheral blood flow; **(b)** left thoracotomy shows the first surgical step where the aorta was bypassed and transected.

As a first stage, after a low-dose heparin infusion, left thoracotomy was performed with the patient in right 60° lateral decubitus. Using an aortic side-clamp to prevent visceral and spinal cord ischaemia, a silver coated dacron aortic graft was anastomosed in an end-to-side fashion to the proximal aorta and distal to the region involved by the tumor. Low-dose heparin was promptly converted after aortic repair without the need for extracorporeal circulation support. The infiltrated aortic segment (8 cm-length) was therefore stapled and transected (Figure 2). The excluded aortic tract was then freed from its segmental intercostal arteries (two couples) and surrounding tissues. During this step, the T7-T8 and T10-T11 disc spaces, corresponding to the caudocranial extension of the following surgical step, were identified and at those levels, a partial discectomy was performed after incision of the longitudinal anterior ligaments. The left 7th-8th-9th ribs were resected in view of the subsequent *en bloc* vertebrectomy. A bovine pericardium patch was finally employed to wrap the excluded structures and prevent adhesions. The early postoperative course was uneventful.

**Figure 3:**
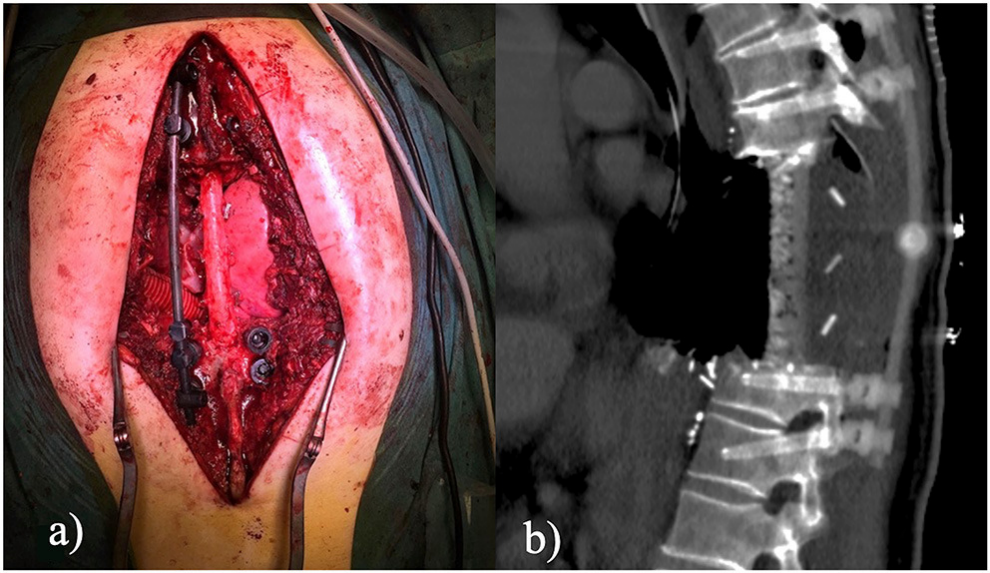
**(a)** Open posterior approach. The figures show the results of a triple *en bloc* vertebrectomy. Pedicle screws were inserted at two levels above and two levels below the pathologic vertebrae; **(b)** Sagittal CT scan. The figure shows the final result after tumor removal and 360° fixation. Instrumentation is radiolucent to consent radiologic follow-up.

Thirteen days later, T8-T9-T10 vertebrectomy was performed. After a midline posterior spine incision, with the assistance of an O-arm and a spine navigator, peduncular screws were placed in T6-T7 and T11-T12. 4.5-mm titanic screws, connected with retractors, were placed to allow the posterior detriment of the T8-T9 and T10 vertebral elements. Spinal reconstruction was performed by placing an expandable cage in PEEK material filled with autologous and synthetic bone. Titanium screws were then replaced with carbon fiber screws (Figure 3).

## Results

**Figure 4:**
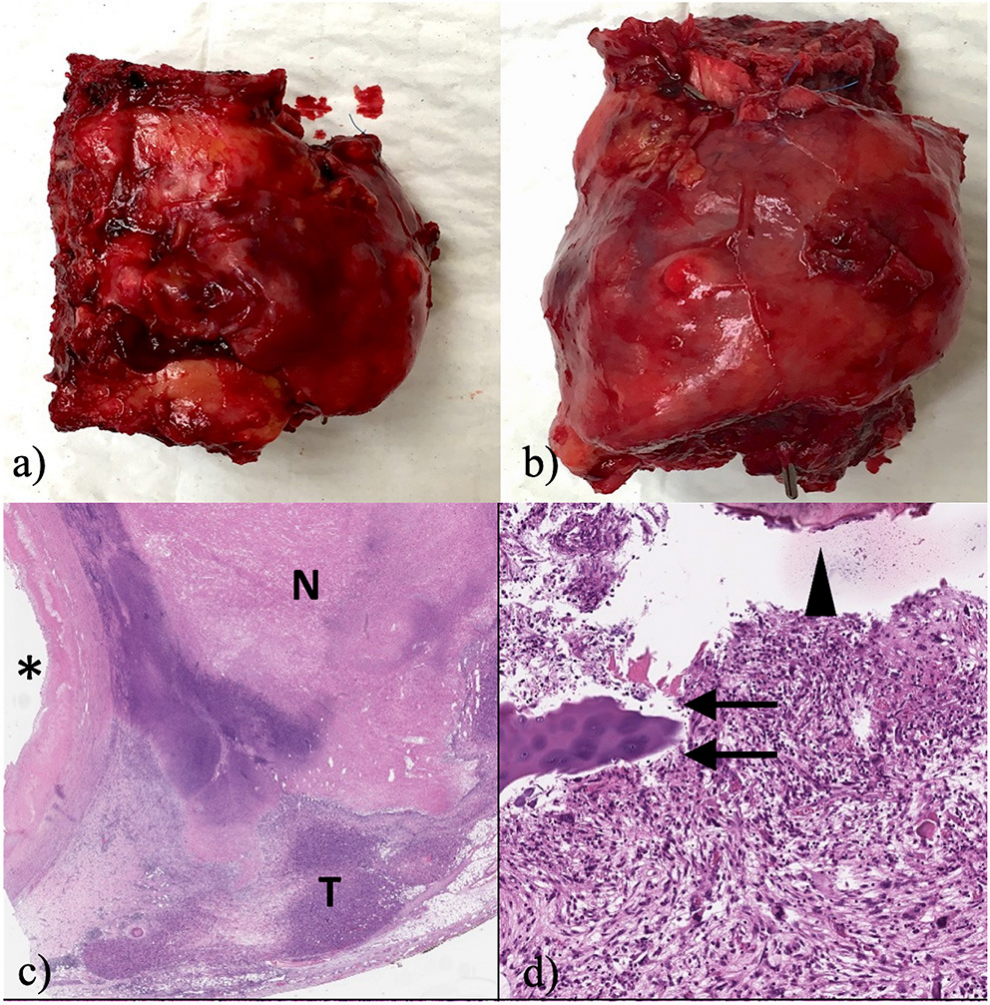
**(a)** Macroscopic lateral view of the resected tumor; **(b)** Macroscopic front view of the resected tumor; **(c)** at scanning magnification of a diffusely necrotic neoplasm infiltrates the adventitia of the aorta (aortic lumen marked by the asterisk; T: indicates the viable tumor; N: indicates the necrotic component; original magnification (OM) 10x; staining: haematoxylin and eosin (H&E); **(d)** depicts bone (arrowhead), cartilage (arrows) and the variable shape and size of the nuclei of pleomorphic soft tissue sarcoma (100x; staining: H&E).

Blood loss was about 2,300 ml. The patient was recovered in the ICU for initial ventilation support and after 48 h was admitted to the ward. No neurological deficits were identified. A dehiscence of the surgical wound, requiring vacuum-assisted closure and antibiotics, was documented. The patient was eventually transferred for rehabilitation after 29 days from the procedure. The resection consisted of three vertebral bodies (7 cm maximum diameter, collectively) and a 5 cm-tract of aorta. The tumor was grossly sized 3.6 cm in maximum diameter and was located between the aorta and the vertebral body, which was fixed to the aortic wall. At cutting section, T9 was soft, mostly non-ossified and fleshy in appearance. The neoplasm showed no immunohistochemistry positivity to alpha-SMA (1A4), p63, CD34, desmin, NGFR, MDM2, myogenin, SOX10, S100 protein, cytokeratin AE1/AE3/PCK26 or EMA; H3K27me3 was expressed in about 10% of tumor cells. Isocitrate dehydrogenase (IDH) genes were not mutated, which excluded the possibility of dedifferentiated chondrosarcoma. The final diagnosis was of undifferentiated pleomorphic sarcoma (UPS), grade 3 FNCLCC (D3M2N2), infiltrating the aortic wall, the soft tissues and the vertebral body of T9. Free margins of resection (R0) were achieved (Figure 4). Unfortunately, the healing of the surgical wound was delayed by local infection, which required protracted vacuum assisted closure (VAC), intravenous antibiotic therapy and finally a surgical revision with plastic surgeon. In particular, the wound presented in his central part cutaneous and subcutaneous ischemic damage without hardware exposure. A muscolar vascularized flap was rotated from dorsal muscle to cover this area, thus solving the complication. Still, these procedures ultimately precluded the scheduled adjuvant therapy.

After 22 months from initial surgery, neither local relapse nor neurological sequelae have been documented.

## Discussion

We reported on an aggressive multi-step upfront surgery that provided mid-term disease-free survival in a locally-advanced paravertebral UPS. In this case, the peculiar location of the primary UPS represented an oncological challenge, as the involvement of the axial skeleton along with the thoracic aorta is extremely infrequent and is associated with poor outcome (4, 5). The MDT's final decision to proceed to an upfront radical resection was supported by several considerations. A neoadjuvant approach such as chemo- and or radiation therapy was discarded, taking into consideration that even a very limited local tumor progression could have hampered radical surgery. Moreover, even a dimensional response would not have modified the surgical approach aimed at oncological radicality. Presurgical aortic irradiation would have weakened the aortic wall, favoring the risk of intraoperative rupture and jeopardizing the endurance of the vascular anastomosis. Finally, to bear such a long and complex intervention, the patient's performance status could not be undermined by neoadjuvant chemotherapy. Tomita et al. described in 1997 for the first time *en bloc* vertebrectomy as a feasible treatment for primary spinal malignancies (6). Since then, only a few cases of combined aortic and vertebral resection have been described (7, 8). To the best of our knowledge, this is the first triple thoracic vertebrectomy combined with an aortic resection reported in the available literature, that was performed without extracorporeal circulation and achieved oncological radicality. Endoluminal aortic enforcement is an alternative and interesting technique to facilitate cancer resection (9). In the case described herein, however, we deemed this strategy inadequate as cancer infiltration involved the right postero-lateral aspect of the aortic wall which is almost, if not quite, not controlled via a left thoracic access.

For such complex and demanding procedures, multi-stage surgery limits morbidity and mortality by reducing operating time, the risk of organ ischaemia, bleeding and infection. In this case, the surgical plan comprised two separate steps. This choice aimed to reduce the overall length of the surgical intervention and to reduce the risk of complications. The decision to postpone the second surgical step from 5–7 to 13 days after the initial procedure was discussed in a multidisciplinary fashion and was finally advocated to assess the success of the grafting procedure, to wait for an optimal patient recovery and to ease spinal reperfusion.

Despite the technical success of the procedure, we acknowledge that a longer follow-up is critical to define the impact of such aggressive surgical resection on local and distant control.

In conclusion, multi-step combined upfront surgery is a feasible and effective strategy to achieve *en bloc* radical resection of unfavorable localization of primary STS. A strategic alliance between multi-speciality surgeons with extensive expertise in STS is mandatory to define an optimal and patient-oriented therapeutic strategy.

## Data Availability Statement

The original contributions presented in the study are included in the article/supplementary material, further inquiries can be directed to the corresponding author.

## Ethics Statement

Ethical review and approval was not required for the study on human participants in accordance with the local legislation and institutional requirements. The patients/participants provided their written informed consent to participate in this study. The patient gave written consent for publication.

## Author Contributions

UC performed surgery designed, supervised, and reviewed the draft. NG, AM, and AB wrote the draft. PN, FC, and EC performed surgery. SR provided histology analysis. All authors contributed to the article and approved the submitted version.

## Conflict of Interest

The authors declare that the research was conducted in the absence of any commercial or financial relationships that could be construed as a potential conflict of interest.

## Abbreviations

UPS: undifferentiated pleomorphic sarcoma
STS: soft tissue sarcoma
^18^F-FDG-PET/CT: 18-fluorodeoxyglucose positron emission tomography/computed tomography
MRI: magnetic resonance imaging
CT: computed tomography
MDT: multidisciplinary team
VAC: vacuum assisted closure.
